# Rheumatoid Arthritis Has Won the Battle but Not the War: How Many Joints Will We Save Tomorrow?

**DOI:** 10.3390/medicina59101853

**Published:** 2023-10-18

**Authors:** Volodymyr V. Oberemok, Oksana Andreeva, Kateryna Laikova, Edie Alieva, Zenure Temirova

**Affiliations:** Department of Molecular Genetics and Biotechnologies, Institute of Biochemical Technologies, Ecology and Pharmacy, V.I. Vernadsky Crimean Federal University, Simferopol 295007, Crimea; andreeva-oksana.94.3@mail.ru (O.A.); botan_icus@mail.ru (K.L.); ediealieva57@gmail.com (E.A.); wwwzzznnn333@gmail.com (Z.T.)

**Keywords:** rheumatoid arthritis, joints, inflammation, antisense oligonucleotides

## Abstract

Rheumatoid arthritis refers to joint diseases of unclear etiology whose final stages can lead to unbearable pain and complete immobility of the affected joints. As one of the most widely known diseases of the joints, it serves as a study target for a large number of research groups and pharmaceutical companies. Modern treatment with anti-inflammatory drugs, including janus kinase (JAK) inhibitors, monoclonal antibodies, and botanicals (polyphenols, glycosides, alkaloids, etc.) has achieved some success and hope for improving the course of the disease. However, existing drugs against RA have a number of side effects which push researchers to elaborate on more selective and effective drug candidates. The avant-garde of research, which aims to develop treatment of rheumatoid arthritis using antisense oligonucleotides along with nonsteroidal drugs and corticosteroids against inflammation, increases the chances of success and expands the arsenal of drugs. The primary goal in the treatment of this disease is to find therapies that allow patients with rheumatoid arthritis to move their joints without pain. The main purpose of this review is to show the victories and challenges for the treatment of rheumatoid arthritis and the tortuous but promising path of research that aims to help patients experience the joy of freely moving joints without pain.

## 1. Introduction

Examination of European and North African skeletal remains has revealed that an-cient people suffered from different forms of arthritis: osteoarthritis, gout and spondy-loarthritis [1,2]. Paleontological analysis of samples dating back several millennia show characteristic features of rheumatoid arthritis (RA) among indigenous tribes in North America [1,3]. A severe climate is a known factor responsible for rheumatoid arthritis [4,5], which may explain these paleontological findings.

One of the first statements in describing the details of RA was recorded in the doctor-al dissertation of the French physician Augustin Jacob Landré-Beauvais [6,7], written in 1800. He defined this symptom and proposed the term aesthetic gout. Alfred Garrod, an English scientist, first used the term ‘rheumatoid arthritis’ in 1859 [8].

Rheumatoid arthritis (RA) is a difficult to treat disease. This disease is characterized by synovial inflammation (synovitis), which affects bone turnover and the ability of bone to adapt to bone tissue when replacing the cartilaginous matrix with mineralized bone [9]; it also leads to degeneration of bone tissue [10]. Patients with this disease experience pain in their joints, accompanied by stiffness, which limits their mobility. In addition, over time, this disease can destroy both cartilage and bone; limited mobility becomes active disability and may lead to deformed joints. Along with deformity, extra-articular manifestations such as vasculitis and scleritis can also be observed [11,12].

Among common risk factors, cigarette smoking has the strongest association with RA [13,14,15,16]. Tobacco smoking, does not cause rheumatoid arthritis but leads to a worsening of the disease by means of citrullination of tissue proteins [17,18]. Interestingly, Jiang et al. reported that the impact of cigarette uses on the development of RA increased only when smoke was inhaled from cigarettes, but not when tobacco products containing nicotine were chewed, which suggests that nicotine is not significantly involved in the pathogenesis of RA [16,17].

As mentioned above, RA is the most common autoimmune inflammatory arthritis, with incidence of 0.5–1.0% in the northern hemisphere population [19,20]; annually, it occurs in 24–45 people per 100,000 [21]. The disability that attends rheumatoid arthritis is serious and debilitating. An extensive review showed that within 2–3 years of onset, approximately one-third of sick people with rheumatoid arthritis stopped working because of the disease; by 10–15 years after onset, almost two-thirds (50–60%) may be unable to work [22,23]. In addition to deterioration of patients’ health, this leads to adverse effects in the global economy [24,25].

The juvenile form of the disease that occupies a significantly higher incidence in comparison with other inflammatory diseases of the joints is a particularly serious problem in children. In some, it may affect vital organs; this, coupled with the inevitable side effects from therapeutic manipulations lead to a deterioration in the health of the entire body [26,27]. Many causes predispose both children and adults to the disease, including heredity, poor hygiene, environmental exposure, poor nutrition, trauma, lactation that lasts a long time, pregnancies, grief, tuberculosis, etc. Among adults, women are more susceptible than men. Worldwide, a large number of organizations share the goals of finding treatments for RA and combating its spread, including the Rheumatology Research Foundation (US) [28], the French Society for Rheumatology (France) [29], and the New Zealand Association of Rheumatologists (NZ) [30] and others.

There is no single cause of rheumatoid arthritis, and its prognosis remains uncertain [31]. But microbiological infections, genetic and environmental factors play a special role in the development of RA. Meta-estimates of the regional prevalence of rheumatoid arthritis in low- or middle-income countries were 0.4% (Southeast Asia), 0.37% (Eastern Mediterranean), 0.62% (Europe), 1.25% (North, Central, and South America), and 0.42% (Western Pacific). A formal meta-analysis cannot be performed for sub-Saharan Africa due to limited data. No significant difference in the prevalence of RA was found between urban and rural areas. While the prevalence of the disease in low- and middle-income men was 0.16%, it climbed to 0.75% in women, a statistically significant difference [32]. Hormones and genetic (X-linked) factors may explain the more frequent occurrence of rheumatoid arthritis among women [33]. For example, estrogens, which control the body’s immune response, can lead to autoimmune diseases [34]. To explain gender bias, scientists have shown that women with RA exhibit non-random X chromosome inactivation (XCI), which can cause autoimmunity. It is believed that this error correlates with the presence of a shared epitope and the duration of the disease. Premature immuno aging, characterized by shorter telomere length, is also associated with the presence of SE [35]. An RA study cohort comprising primarily women (77.1%) demonstrated the tendency of the disease to affect middle-aged women; in this study, the median age at diagnosis was 58.7 years and at death was 74.8 years [36]. The mortality risk for patients of either sex with RA is higher than for the general population. Previous studies have confirmed a mortality rate 1.29- to 2.03-fold higher. That being said, while the specific cause of death differs from country to country [37], the main causes of death included an increased incidence in circulatory system, oncological, and respiratory system diseases [36,38]; dementia; and diabetes [39].

## 2. A Mystery That Still Needs to Be Deciphered

Although assimilation of knowledge in the study of pathogenesis of RA is still incomplete, this disease is widely accepted as an immune-mediated disorder. That immune cells play an important role in the pathogenesis of RA has been shown by studies in which treatment with anticytokine agents, for example a neutralizing Abs against TNF-α, soluble TNF receptor fusion proteins, or rIL-1β receptor antagonists resulted in the successful suppression of joint inflammation [40]. Agents that target cytokine-driven immune processes are among the most important clinical treatments used to manage disease in RA patients [41]. Disease-modifying antirheumatic drugs (DMARDs), which are divided into synthetic (sDMARDs) and biological (bDMARDs), are widely used [42]. There are also new targeted synthetic disease-modifying antirheumatic drugs (tsDMARDs) such as JAK inhibitors [43], IL-6R inhibitors [44], and anti-CD20 antibodies [45]. TsDMARDs are small molecules that provide protection against pro-inflammatory cytokines, compared to bDMARDs which can block specific extracellular molecules [46]. However, future studies are needed to monitor the risk-benefit ratio, given the increased risk of infectious diseases and thromboembolism, among others. Now, every year there are new medicines that are able to fight this insidious disease. And although there is no way to completely cure rheumatoid arthritis at this time, the goal of treatment implies remission and reduction of side effects [47,48,49]. In addition, international guidelines on management have been developed that improve the quality of treatment of the disease, based on such treatment principles as a strict control strategy and a targeted approach to treatment [50].

There are three pronounced determinants that can describe the clinical picture of RA: the inflammatory process (swelling, pain, stiffness during movement); the proliferative-destructive process (destruction of joints); and the enzymatic collagenolytic process (primary necrotism) [51]. The intensity of pain is directly related to the activity of the disease as a whole [52]. The pathogenesis of RA has different etiologies of origin. Since the early 1980s, it has been assumed that the spread has a genetic link [53], including exposure to various chemicals on the respiratory tract [54]. There are a variety of ways to counteract RA, and one of the first measures is dietary nutrition, since there are already a number of scientific justifications that show an advantage in combating the disease and lead to a reduction in symptoms [55].

The pathogenesis of the studied disease involves a complex interconnection between B cells, CD4+ and CD8+ T cells, and dendritic cells [56]. Because B cells undergo isotype switching, they are more capable of supporting the inflammatory cascade. In addition, the rheumatoid factor (RF), a group of autoantibodies with the ability to respond to Fc of human IgG, behaves like heterophilic antibodies and cross-reacts with other types of antibodies [57,58]. The participation of the RF in the formation of the immune complex can lead to further fixation of the complement and the involvement of cells that cause inflammation such as macrophages, neutrophils, and lymphocytes. This leads to tissue damage and provides positive feedback for the production of even more autoantibodies. At the moment, a long list of causes for rheumatoid arthritis has been identified, including epigenetic, genetic, hormonal, reproductive, neuroendocrine and comorbid host factors [59].

It appears that macrophage-derived cytokines, for example, tumor necrosis factor alpha (TNF-α) and IL-1β, are critical to the mediation of inflammatory synovitis; however, it has also been suggested that synovial T cells participate both in triggering the disease and in contributing to the development of the disease in RA. Significant infiltration of the synovial tissue by T lymphocytes has been observed frequently in RA [60]. However, little is known about the direct action of T cells in the development of pathogenesis. While the synovial T cells observed in RA are mainly classified by type of memory type: CD4+ CD45 RO [61] with a pro-inflammatory Th1 phenotype. Compared to TNF-α and IL-1β, the classical Th1 T-cell-derived cytokine IFN-γ is also rarely seen in rheumatoid joints [62].

Scientists believe that T cells-produced cytokine IL-17 participates in the RA development. IL-17 is frequently produced by T cells clones taken from patients with RA, and it was shown that IL-17 is found in abundance in the synovial fluids of RA patients [63]. Therefore, it comes as no surprise that T cells specifically predisposed to antigen have been shown to be efficacious in the medication of rheumatoid arthritis. Generally, these data suggest that T cells are quite significant albeit their still incompletely understood role in the pathogenesis of RA.

A typical ‘Western’ diet, rich in calories and insufficient in fiber, increases the risk of disease [64]. However, the intake of omega-3 fatty acids has been led to a reduction in risk of RA. Investigation of the link between diet and immune diseases has shown that nutritional factors can function as environmental triggers in genetically predisposed individuals. In RA, these triggers set in motion a cascade of events [65] that includes the elaboration of chemokines and cytokines (soluble immune mediators) by cells of joint tissue, such as synovial macrophages, synovial fibroblasts, and chondrocytes, resulting to joint damage and deformity [64,66].

Recently, we began to better understand the etiology of this disease [67]. For instance, we have learned that RA is a heterogeneous disease that, according to the data combining genetic risk factors and autoantibodies, can be classified positive and negative for anti-citrullinated protein antibodies (ACPA). Some critical immune responses manifest very early in RA patients. For example, elevated C-reactive protein levels, ACPA, and RF are detected in some patients years before the clinical symptoms are seen [68].

Studies have been conducted based on function enrichment analysis, which shows that the RA-related modules were significantly enriched in immune-related actions [69]. Hub genes were then identified as candidate genes. This analysis of scientists showed that the expression levels of candidate genes are significantly associated with the immune microenvironment of RA, which in the future may lead to earlier detection of RA disease. A number of other studies have reported that expression quantitative trait loci (eQTL) analysis has revealed dynamic variations in eQTL effects in the context of immunological conditions as well as cell types. These cell type-specific and context-dependent eQTLs showed significant enrichment in genetic variants associated with immune diseases, and they affect cell types, genes and environments associated with disease [70].

It has also been proven that an increase in pre-DC (dendritic cell precursors) in peripheral blood predicted RA treatment resistance. Pre-DC could have patho-physiological relevance to RA treatment response [71].

Moreover, the identification of important cytokine signaling pathways taking part in disease progression along with synovial studies have demonstrated the importance of both adaptive and innate immune responses [72]. The increase in knowledge concerning the pathophysiology of this disease has significantly expanded specific treatment options. Currently we possess a wide range of therapeutic options ranging from established drugs such as steroids and disease-modifying anti-rheumatic drugs to targeted therapies that aim to inhibit particular cells or cytokines [73].

The introduction of drugs that inhibit pro-inflammatory cytokines heralded a significant shift in the progress of the treatment of RA, particularly those that target (TNF-α). Measurement of the amount of this cytokine in the peripheral blood of patients with RA has been used to assess the severity of the disease [74].

## 3. Achieving Movement without Pain

In addition to adalimumab, drugs such as methotrexate [75], etanercept [76,77], prednisone [78], and leflunomide [79] quite often used in the treatment of RA. These drugs have been in use longer, and for a variety of conditions. Their side effects are also serious and must be considered.

According to EULAR (European alliance of associations for rheumatology) recommendations the biologic and targeting synthetic drugs should be used only after treatment with methotrexate [80]. Methotrexate is the first-choice treatment among most patients [81]. Treatment with methotrexate reduces inflammation but also degrades cartilage. It is unknown whether these clinical responses to methotrexate are evidence of a specific mechanism of action or merely a common final pathway. While it is possible that changes in the production of PGE2 and levels of TIMP 1 may represent the downstream effects of methotrexate on the formation of IL-1 and IL-6, respectively, no conclusive evidence exists to support this. Some of these changes are also observed following treatment with nonsteroidal anti-inflammatory drugs, and the methotrexate-specific pathways have yet to be elucidated [82]. In addition, the number of side effects—some severe—associated with methotrexate are many: nausea, headaches, fatigue, mucositis and hair loss, cytopenia, interstitial lung disease (ILD pneumonitis), and drug-related liver diseases (fibrosis and cirrhosis of the liver) [83]. Unfortunately, the exact mechanisms of methotrexate toxicity remain unclear [84] (Table 1).

Prednisone is another drug used to treat RA. It is a corticosteroid with anti-inflammatory properties and immunosuppressive activity [91,97]. The mechanism of action is the binding of prednisone to the glucocorticoid receptor; it promotes conformational changes in the DNA-binding domain, which leads to a displacement of the receptor into the nucleus. In the latter, various genes are activated, including anti-inflammatory genes [91]. Among these are the genes encoding annexin-1 (formerly known as lipocortin 1), IαB (NFαB inhibitor), IL-10, and the anti-inflammatory protein MAPK-phosphatase-1. This process, called transactivation, is responsible in part for the anti-inflammatory action of glucocorticoids. Another mechanism involved in the anti-inflammatory process is transrepression, in which glucocorticoids prevent the interaction of transcription factors such as AP-1 and NFαB with DNA, thereby eliminating the formation of pro-inflammatory cytokines. This transactivation and transrepression inhibit the formation of inflammatory mediators, supporting the powerful anti-inflammatory effect of glucocorticoids [98].

The undesirable effects of corticosteroids are doses and time dependent and vary depending on the drug administered [99]. Some adverse effects follow a linear dose-response pattern, where the incidence increases together with dose increases (ecchymosis, cushingoid features, parchment-like skin and sleep disturbance). Other adverse effects may instead ensue a threshold dose-response pattern, where the probability of the disease only becomes elevated beyond a distinct threshold value (weight gain and epistaxis at prednisone doses greater than 5 mg daily, glaucoma, depression, hypertension at prednisone doses greater than 7.5 mg daily, etc.) [92].

Patients with RA are often prescribed anti-rheumatic drug leflunomide whose primary aim is to reduce swelling and inflammation in the affected joints [97]. The active metabolite of leflunomide (teriflunomidum, or A77 1726) in reversible manner inhibits dehydroorotate dehydrogenase, a step limiting the rate of de novo pyrimidine synthesis [93]. This results in a reduction in the level of circulating pyrimidines and affects their availability for DNA and RNA synthesis, which in turn has an effect on the spreading of immune cells as well as the expression of inflammatory cytokines. Leflunomide inhibits the capacity of T lymphocytes to promote monocytes through direct cell-cell contact in vitro.

Results from a Phase II clinical trial of leflunomide in patients revealed that A77 1726 binds to plasma protein with great efficiency (>99%) [100]. In studies conducted on animal models, leflunomide has been demonstrated to be extremely effective in the treatment of both adjuvant [101,102] and collagen induced arthritis [93,103]. The leflunomide metabolite A77 1726 adjusts lymphocyte proliferation both in vitro [104] and in vivo [105] (it is an equally efficacious immunoregulator of CD4+ and CD8+ T cell proliferation when induced by mitogens mediated by cell surface receptors). Currently, two specific mechanisms of action have been found for A77 1726: inhibition of tyrosine kinases and de novo inhibition of pyrimidine nucleotide biosynthesis at the end of G1 (growth). The most common undesirable effects of leflunomide treatment were gastrointestinal disorders (diarrhea), elevated liver function tests, abdominal pain, nausea/vomiting, allergic reactions, and reversible alopecia [94,95,96].

Due to the highly complex interaction among genetics and epigenetics, truly individual therapy for RA is impossible at this time. Currently, standard therapeutic algorithms are used, but they are unable to consider the individual characteristics of the patient [73].

Difficulties arise in treatment and prevention; for example, it is not always possible to control the degree and activity of the disease, despite the use of several drugs with different mechanisms of action. The type of RA, which is difficult to treat, is called D2T, which is a heterogeneous and multifactorial disease [106,107].

## 4. Botanicals as a Beacon of Hope on the Horizon

Herbal preparations used in humans demonstrate their effectiveness in the treatment of rheumatoid arthritis (Table 2). One of the main positive effects is anti-inflammatory and antioxidant activity [108,109,110]. For example, such an herb as *Boswellia* spp., which has been used in Ayurvedic medicine since ancient times. Activity of boswellic acid and other active natural compounds of *Boswellia* spp. includes inhibition of microsomal prostaglandin E2 synthase-1 (PGE2) and 5-lipoxygenase, reducing the production or activation of inflammatory mediators such as matrix metalloproteinase (MMP)-9, MMP-13, cyclooxygenase (COX)-2, and nitric oxide (NO) and also has analgesic and anti-arthritic effects [104,105]. It has been found to reduce the number of osteophytes by attenuating inflammatory mediators such as C-reactive protein and hyaluronic acid [111,112,113]. Research by scientists has demonstrated the safety of using *Boswellia serrata* R. [112]. However, knee-related activities of daily living and quality of life did not improve significantly [114].

Polyphenols constitute an alternative direction of using natural compounds against rheumatoid arthritis. Application of an extract from *Curcuma* spp. containing polyphenol curcumin also showed its anti-inflammatory and antioxidant actions [115,116]. Curcumin inhibits the production of inflammatory mediators, such as a variety of MMPs, tumor necrosis factor-alpha (TNF-α), interleukin (IL)-8, IL-1, NO, via diminishing the activation of MAPK signaling pathways protein kinase B (Akt) and NF-κB [117,118] and leads to a decrease in prostaglandin synthesis [119]. Studies have shown that when compared with the treatment of ibuprofen, turmeric showed more effective results [120], and compared with diclofenac, a small number of side effects [121]. The dried plant *Matricaria chamomilla* L. has been used for many centuries in the treatment of joint pain [122,123]. The plant contains several phenolic compounds: apigenin, patuletin, luteolin, and glycosides [124] that reduce inflammation by reducing the levels of cytokines and PGE2, which play a role in the pathogenesis of arthritis. Epigallocatechin-3-gallate (EGCG), a catechin monomer that has antioxidant and anti-inflammatory effects, is isolated from tea leaves [125]. Green tea extract can inhibit the expression of IL-1β-induced chemokines This was studied in an experiment with rats having arthritis [126,127].

Date seeds of *Phoenix dactylifera* L. are a well-known traditional Moroccan remedy for pathological conditions involving inflammation such as RA [128]. The wild pomegranate *Punica granatum* L. has been used as a traditional medicine for various conditions, including pain and inflammation [129]. Pomegranate demonstrated potential inhibition of NO as well as reduction in paw edema in carrageenan-induced mice after administration of 100 mg/kg [130]. Pomegranate juice is one of the natural products that has also shown promising results in clinical trials for the treatment of RA symptoms, which may also be due to polyphenolic compounds with antioxidant and anti-inflammatory effects [131].

Glycosides from different plants show anti-inflammatory effect during rheumatoid arthritis. The root of *Paeonia lactiflora* P. has been used in Chinese medicine since ancient times. Inhibition of the production of leukotriene B4, PGE2, ROS, NO, and other pro-inflammatory mediators by paeoniflorin and total glucosides of paeony has been proven [132]. Combined treatment using this plant and methotrexate has shown beneficial effects in RA with fewer side effects [133]. Decoctions from *Eremostachys laciniata* (L.) are also used for arthritis [134,135,136]. It is assumed that the iridoid glycosides of this plant exhibited an anti-inflammatory effect. *Curculigoorchioides* G. contains curculiglycoside, which improves arthritis symptoms in rats [128] induced by collagen type II (CIA) and reduces levels of inflammatory factors (TNF-α, IL-1β, IL-6, IL-10, IL-12 and IL-17A). Its antiarthritic molecular mechanism may be related to the JAK/STAT/NF-Κb signaling pathway [137]. Extracts from the root of *Tripterygium wilfordii* Hook F. also has an immunosuppressive effect and inhibits the expression of pro-inflammatory mediators and cytokines, adhesion molecules and matrix metalloproteinases by macrophages [138,139,140]. However, adverse reactions have been reported in the form of episodic severe toxicity [141,142,143].

Alkaloids of some plants show promising results against rheumatoid arthritis too. Synomenine, which is contained in the Chinese medicinal stem *Sinomenium acutum* Thunb., is used in the treatment of rheumatic diseases [144,145]. Synomenine can phosphorylate p62 Ser351, degrade Keap1 and increase Nrf2 expression, and play a role in protecting against bone destruction by increasing p62 expression and activating the p62-Keap1-Nrf2 axis. Research shows that sinomenine has an immunoregulatory effect on RA [146,147,148]. Dry root of *Aconite kusnezoffii* Reichb. (caowu) has been used for many years to treat RA and relieve joint pain due to its anti-inflammatory properties. Pharmacological studies have shown that diterpenoid alkaloids (mesaconitine, hypaconitine, neolin, talatizamine) are responsible for the main biologically active effects of *A. kusnezoffii* and provide a promising strategy for RA therapy [149,150]. The Radix Linderae, the dry roots of *Lindera aggregata* (Sims) Kosterm. contain the alkaloid norisoboldine (NOR), which has anticancer activity [151]. Also, studies [152,153] have shown that NOR can inhibit bone and cartilage destruction in antigen-induced arthritis (AIA) rats by downregulating the expression of RANKL, IL-6, PGE2, and MMP-13 via the p38/ERK/AKT/AP-1 pathway.

**Table 2:** Common botanical remedies used to treat and prevent RA.

Plant	Mechanism of Action	Appearance
*Boswellia* spp.	Inhibition of microsomal prostaglandin E2 synthase-1 (PGE2) and 5-lipoxygenase [104,105].	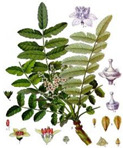
*Curcuma* spp.	Inhibits the production of inflammatory mediators (MMPs), tumor necrosis factor-alpha (TNF-α), interleukin (IL)-8, IL-1, NO [117,118] and leads to a decrease in prostaglandin synthesis [119].	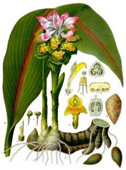
*Punica granatum* L.	Inhibition of NO as well as reduction in paw edema in carrageenan-induced mice after administration of 100 mg/kg [130].	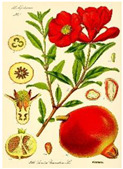
*Paeonia lactiflora* P.	Inhibition of the production of leukotriene B4, PGE2, ROS, NO [132].	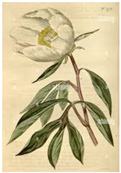

In recent years, scientists have agreed that a mixed herbal decoction can actively manifest itself in the fight against RA. For example, wutou decoction which consists of ephedra, peony, astragalus, licorice and Sichuan aconite [154]. In a study, wutou decoction was shown to effectively inhibit the expression of iNOS, TNF-α, and IL-6. Another example would be GuiZhiShaoYaoZhiMu Decoction (GSZD), which consists of *Ramuluscinnamomi*, *P. lactiflora root*, Radix GlycyrrhizaePreparata, *Ephedra* sp., *Anemarrhenaasphodeloides Bunge root*, *Atractylodesmacrocephala* and *Zingiber officinale* [155,156]. Combination treatment with GSZD and methotrexate was more effective and safer than RA treatment with methotrexate alone [157].

Thus, there is strong evidence that botanicals can help with rheumatoid arthritis, triggering anti-inflammatory and antioxidant activity and decreasing or stopping the speed of biochemical reactions, leading to less pronounced disease. The main disadvantage is that it requires long-term treatment, especially when achieving the goal of pain relief, and adverse reactions can occur [158,159]. Nevertheless, glycosides, polyphenols, alkaloids and other natural compounds produced by plants are very helpful and can strengthen the effect of traditional drugs, or even substitute them in some conditions.

As herbal extracts and nutritional supplements become more popular, research on the potential benefits of herbal supplements for arthritis is growing [160,161]. Modern pharmacological preparations make it possible to relieve the pain and symptoms of RA, as well as improve the quality of life. Concerns about the safety and cost of traditional arthritis treatments have fueled interest in natural remedies. In addition, the difficulty in treating chronic arthritis pain has led to research into herbal therapies. Herbs may offer a complementary or alternative method of effective and safe treatment [162]. The use of herbal medicines among arthritis patients is on the rise, and around 60–90% are expected to seek complementary and alternative medicine options.

**Figure 1:**
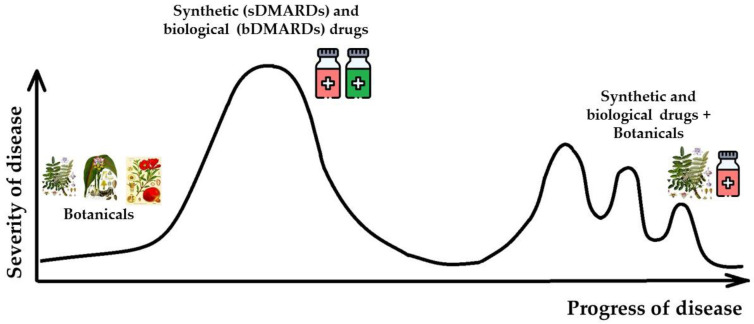
Possible scheme for the use of anti-rheumatoid preparations in different phases of disease development.

In our opinion, the use of botanicals preparations is appropriate in early stages of the disease or when a period of remission begins. In the latter case, botanicals are best used in combination with synthetic and biological drugs (Figure 1). On the contrary, solely synthetic and biological drugs are better in acute stages of the disease.

There is a widespread belief among scientists that herbs and their extracts can provide a safe and fairly effective complementary therapeutic approach to the treatment of rheumatoid arthritis [163,164]. Scientific research proves that from a pharmacological point of view, natural plant extracts or mixed plant compounds effectively regulate the human immune system to alleviate RA by inhibiting pro-inflammatory cytokines [165].

## 5. But How Many Joints Will We Save Tomorrow?

Science is always moving forward and actively developing, making new methods of treatment available to human beings.

Today, different approaches exist that help reach successful regeneration, ranging from a gene-manipulated stem cell laden scaffold for cartilage regeneration [166] to a material-free cell therapy [167]. A promising strategy in medicine is the use of antisense techniques. Antisense technology has been widely and thoroughly explored. The perspective of antisense technology is that specific DNA or RNA can connect to target mRNA and afterward turn the ‘undesired’ gene off. According to complementarity principle, designed DNA or RNA molecules target mRNA of interest, effectively resulting in the degradation of the target mRNA or blocking initiation of translation [168,169].

Oberemok’s research group conceptualized and conducted research with the phosphorothioate antisense oligonucleotide (ASO) Cytos-11 that targets TNF-α mRNA. Cytos-11 selectively lowered levels of TNF-α in the peripheral blood and reduced swelling of joints in rats with RA with an efficacy similar to that of adalimumab. Generally, Cytos-11 was well tolerated by the rats, showing a low frequency of immunological reactions. Obtained results showed the potential of ASO to be used in combination with other drugs or as a monotherapy [170].

In an attempt to develop oligonucleotide therapy aimed at synovitis (an inflammatory disease of the synovial membrane of joints or ligaments with the accumulation of inflammatory effusion in the cavity), modulation of the phenotype of activated proliferative inflammatory synovial fibroblasts using antisense oligonucleotides was documented. For example, antisense oligonucleotides developed by Nakazawa et al. targeting the Notch-1 protein have been reported to inhibit both basal and TNF-α-caused proliferation of human synovial fibroblasts isolated from the synovial membrane of a patient with RA [171,172]. It has also been reported that antisense knockdown of the PTPN11 gene that encodes SHP-2 (a known proto-oncogene) inhibits the migration and survival of synovial fibroblasts [171,173]. Consequently, oligonucleotides targeting lncRNAs, which interfere with regulation in the tissues of an arthritic joint, can provide new therapeutic strategies triggering epigenetic factors involved in joint inflammation [171,174]. It is important to note that since oligonucleotide therapy is based on a particular gene sequence, it is expected that oligonucleotides will work specifically on the target gene and may thereby be less prone to causing non-targeted effects or adverse side effects. Actually, the same basic chemical composition of oligonucleotides and the safety profile determined for oligonucleotide therapy in the clinic show evidence that the failure of late-stage clinical trials with drugs of this class may be less common than with monoclonal antibodies [171].

In another study carried out using human cells, a 20-mer-modified ASO with methoxyethyl protection (ISIS 104838) has been investigated, which showed pronounced efficacy, good tolerance, and drug stability during Stage 1 clinical trials. Its pharmacological effect was found to be a dose-dependent, linear, specific reduction in the synthesis of TNF-α by leukocytes in peripheral blood after stimulation with lipopolysaccharide ex vivo; in addition, the highest concentration of ASO in the plasma proportionally and predictably coincided with the dose [170,175].

To us, an additional advantage of antisense technologies in the fight against RA is that treatment with antisense oligonucleotides shows great promise in ending the unbearable pain that accompanies rheumatoid arthritis. These oligonucleotides have proven to be effective pain blockers in other different diseases: nerve injury-induced neuropathic pain [176], spinal muscular atrophy and severe infantile neurological disorder [177], and postoperative pain [178].

In the future, better treatment for rheumatic diseases is indeed possible through the use of new methods of genetic engineering or cell therapy, such as autologous stem cell transplantation [179,180] and CAR-T-cell therapy [179,181]. Although these therapies are currently still risky and costly, the need for effective methods of therapy for autoimmune diseases is urgent, which is why other avenues should be explored as well. In the future, a new goal may be to find a cure, not just remission, for the disease [179].

While there is currently no cure for RA, the treatment aims to expedite diagnosis and promptly achieve a state of low disease activity [182]. Vaccinations may enable the early prevention of RA. Recently, immunization with the protein 14-3-3zeta (ζ), which is involved in T-cell polarization and IL-17A signal transduction, has been shown to suppress arthritis in 14-3-3ζ knockout inflammatory arthritis rat models by the suppression of IL-1α levels and amplified collagen production [183]. In another approach, peptide vaccine CEL-4000 utilizes an MHC class-II specific ligand to activate regulatory responses and subsequently trigger the polarization of T helper cell 2 (Th2) [184].

Another promising approach, DEN-181 immunotherapy, introduces liposomal technology to inject collagen II as a liposome-encapsulated antigen together with calcitriol in patients with ACPA-positive RA. Results from a Phase 1 trial have shown that in addition to its effect on antigen-specific T-cells in RA patients, DEN-181 established a good safety profile. Any specific effects of DEN-181 on the prevention of RA will be investigated in future trials [73].

Great progress has been made in the field of disease monitoring and diagnostics. Potential biomarkers or cytokine panels promise to allow earlier diagnosis and treatment monitoring tracking [185]. With ever-improving high-resolution imaging techniques and the widespread availability of ultrasound, RA can be found at a very early stage, allowing even minor disease progression to be assessed [73].

Funds have already begun to appear for predicting the outcome of therapy and treatment of the disease [186]. With the help of artificial intelligence, it is possible to investigate changes in arthritis using X-rays [187] and can be used in silico trials to develop new treatments [188]. These new technologies using computerization will help to make more accurate predictions for patients for the future. The P4 (predictive, preventive, personalized and participatory) medicine approach has also appeared for early diagnosis of the disease and prevention [189].

Thanks to innovative methods, new opportunities appear in the fight against rheumatoid arthritis. Cuproptosis is an innovative method of treatment of rheumatoid arthritis, which is based on the application of the principle of pneumatic compression of the air bag using special equipment. First, cuproptosis in multiple immune cells may be suppressed, and this suppression contributes to their over-proliferation in RA. Secondly, several essential regulatory genes of cuproptosis have been identified to be associated with multiple RA processes, such as aberrant fibroblast-like synoviocytes (FLS) proliferation and inflammatory processes in various immune cells. Cuproptosis is a safe and non-invasive method of treatment that does not require the use of medications or surgical interventions. Well-designed preclinical experiments and clinical trials are still required for in-depth studies of cuproptosis and its associated genes in the context of RA, which still present a significant challenge. However, it is a research direction with great potential [190].

Another innovative method is the use of gene therapy technology. An innovative method of treating rheumatoid arthritis using histone modification is a reliable alternative to traditional approaches. Histones are proteins that pack DNA inside a cell and regulate gene activity. Histone modification consists of changing the chemical structure of these proteins, which allows you to control the activity of certain genes. Found that in PDGF-induced FLS, the expression of Jumonji C family of histone demethylases (JMJD3) was increased through the Akt signaling pathway, and the proliferation and migration ability of FLS was weakened after inhibition or silence of JMJD3, and the symptoms of DBA/1 mice by collagen-induced arthritis were alleviated [191]. Recent research has shown that histone modifications may be involved in the development and progression of RA. Studies have found that specific histone modifications, such as histone acetylation and methylation, are associated with increased inflammation and joint destruction in RA [192].

In recent years, a method of treating rheumatoid arthritis based on the use of phosphates has been developed. Studies have shown that phosphates can have an anti-inflammatory effect by reducing the production of inflammatory cytokines. They are also able to improve the function of the immune system. Dexamethasone sodium phosphate (DSP) is another anti-inflammatory and immunosuppressive glucocorticoid known to be used frequently in treating RA by decreasing cytokines expression and impeding functions of leukocyte, fibroblast and endothelial cells. Delivered using the beta-cyclodextrin nanocarriers (DSP-loaded H-βCD nanoparticles) was effective for RA in the AIA rat model. Thus, the use of nanocarriers has overcome the limited applications of DSP alone in chronic diseases and has been reported to be able to reduce arthritic score, paw thickness, and cytokine level [193].

**Figure 2:**
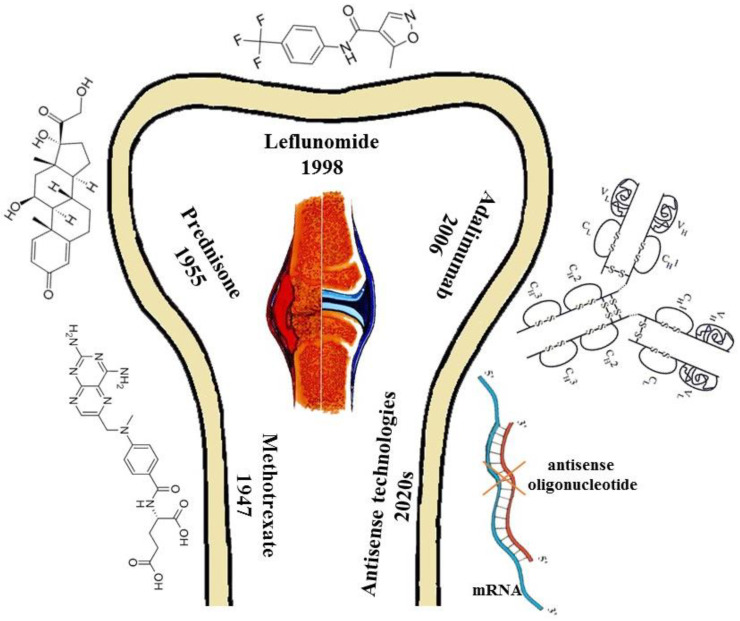
The evolution of medicines for the treatment of rheumatoid arthritis: beginning from methotrexate to prednisone, leflunomide, adalimumab and ending with antisenseoligonucleotide.

It is obvious that today medical preparations have achieved better results in the treatment of RA (Figure 2). However, herbal preparations also have potentially high prospects but have not yet been fully disclosed. Joint application of both botanicals and medical preparations, may bring us to new era of RA treatment, safe and efficient for patients.

## 6. Conclusions

Rheumatoid arthritis does not kill quickly but today it looks like a sentence to a hard-to-treat disease. This, coupled with the many different factors that can trigger RA, may explain why this disease is not treated successfully, with continued high incidence. Now the range of drugs is extensive, and nevertheless has its negative sides. Namely, side effects such as nausea, headaches, fatigue, allergic reactions, and hair loss.

A beacon of hope on the horizon is the use botanicals (polyphenols, glycosides, alkaloids, etc.) and antisense technologies, which have already proven themselves in a number of studies on the treatment of rheumatoid arthritis [194], as well as on the relief of pain in the treatment of diseases such as neurological dysfunction.

Two other useful proactive strategies are vaccination and early diagnosis of the disease, both of which can help prevent and forestall the development of any disease that has already begun. As far as treatment of ongoing disease, the ideal drug has not yet been found, and all of those in use have their side effects. But the prospects for the treatment of rheumatoid arthritis, despite its tortuous solutions, are bright, with promising areas of research fueled by people determined to help these patients. Though today’s battle is lost to rheumatoid arthritis, humanity prepares its joints for the win in the war for movement without pain.

## Figures and Tables

**Table 1:** Comparative characteristics of drugs against RA.

Drug	Characteristic	Mechanism of Action	Side Effects
Adalimumab	A blockbuster product based on monoclonal antibodies.	The inhibition of TNF-α	Including the risk of developing serious infections, particularly of the lungs [85,86,87], the development of deep fungal infections [88,89].
Methotrexate	FDA approved folic acid antagonist [90].	Inhibits enzyme AICAR transformylase; acts as an antifolate antimetabolite; leads to suppression of T cell activation, downregulation of B cells, increased sensitivity of activated CD-95 T cells.	Nausea, headaches, fatigue, mucositis and hair loss, cytopenia, ILD pneumonitis, and drug-related liver diseases (fibrosis and cirrhosis of the liver).
Prednisone	Corticosteroid with anti-inflammatory properties.	Promotes conformational changes in the DNA-binding domain, which leads to a displacement of the receptor into the nucleus [91]; action is the binding of prednisone to the glucocorticoid receptor, it promotes conformational changes in the DNA-binding domain, which leads to a displacement of the receptor into the nucleus; anti-inflammatory process is transrepression, in which glucocorticoids prevent the interaction of transcription factors.	Ecchymosis, cushingoid features, parchment-like skin and sleep disturbance [92].
Leflunomide	Anti-rheumatic drug, (teriflunomidum, or A77 1726) [93].	In reversible manner inhibits dehydroorotate dehydrogenase [93].	Gastrointestinal disorders (diarrhea), elevated liver function tests, abdominal pain, nausea/vomiting, allergic reactions, and reversible alopecia [94,95,96].

## References

[1] McInnes I.B., Schett G. (2011). The pathogenesis of rheumatoid arthritis. N. Engl. J. Med..

[2] Aceves-Avila F.J., Baez-Molgado S., Medina F., Fraga A. (1998). Paleopathology in osseous remains from the 16th century. A survey of rheumatic diseases. J. Rheumatol..

[3] Rothschild B.M., Turner K.R., DeLuca M.A. (1988). Symmetrical erosive peripheral polyarthritis in the Late Archaic Period of Alabama. Science.

[4] Zeng P., Bengtsson C., Klareskog L., Alfredsson L. (2017). Working in cold environment and risk of developing rheumatoid arthritis: Results from the Swedish EIRA case—Control study. RMD Open.

[5] Huang L.J., Zha J.J., Cao N.W., Zhou H.Y., Chu X.J., Wang H., Li X.B., Li B.Z. (2022). Temperature might increase the hospital admission risk for rheumatoid arthritis patients in Anqing, China: A time-series study. Int. J. Biometeorol..

[6] Landre-Beauvais A.-J. (1800). Doit-on Admettreune Nouvelle Espece de Goutte sous las Denomination de Goutte Asthenique Primitive?. Doctoral Thesis.

[7] Bannatyne G.A., Wohlmann A.S., Bladall F.R. (1896). Rheumatoid arthritis: Its clinical history, aetiology and pathology. Lancet.

[8] Symmons D.P. (1995). What is rheumatoid arthritis?. Br. Med. Bull..

[9] Berardi S., Corrado A., Maruotti N., Cici D., Cantatore F.P. (2021). Osteoblast role in the pathogenesis of rheumatoid arthritis. Mol. Biol. Rep..

[10] Cheng C., Liao H., Wu C. (2022). Tissue microenvironment dictates inflammation and disease activity in rheumatoid arthritis. J. Formos. Med. Assoc..

[11] Go F.G., Midwood K.S. (2011). Internal danger: Activation of Toll-like receptors in rheumatoid arthritis. Rheumatology.

[12] Klarenbeek N.B., Kerstens P.J., Huizinga T.W., Dijkmans B.A., Allaart C.F. (2010). Recent advances in the management of rheumatoid arthritis. Br. Med. J..

[13] Chauhan K., Jandu J.S., Brent L.H., Al-Dhahir M.A. (2023). Rheumatoid Arthritis.

[14] Sugiyama D., Nishimura K., Tamaki K., Tsuji G., Nakazawa T., Morinobu A., Kumagai S. (2010). Impact of smoking as a risk factor for developing rheumatoid arthritis: A meta-analysis of observational studies. Ann. Rheum. Dis..

[15] Makrygiannakis D., Hermansson M., Ulfgren A.K., Nicholas A.P., Zendman A.J., Eklund A., Grunewald J., Skold C.M., Klareskog L., Catrina A.I. (2008). Smoking increases peptidylarginine deiminase 2 enzyme expression in human lungs and increases citrullination in BAL cells. Ann. Rheum. Dis..

[16] Ishikawa Y., Terao C. (2020). The Impact of Cigarette Smoking on Risk of Rheumatoid Arthritis: A Narrative Review. Cells.

[17] Alsalahy M.M., Nasser H.S., Hashem M.M., Elsayed S.M. (2010). Effect of tobacco smoking on tissue protein citrullination and disease progression in patients with rheumatoid arthritis. Saudi Pharm. J..

[18] Jiang X., Alfredsson L., Klareskog L., Bengtsson C. (2014). Smokeless tobacco (moist snuff) use and the risk of developing rheumatoid arthritis: Results from a case-control study. Arthritis Care Res..

[19] Smolen J.S., Aletaha D., McInnes I.B. (2016). Rheumatoid arthritis. Lancet.

[20] Yin X., Cheng F., Wang X., Mu J., Ma C., Zhai C., Wang Q. (2019). Top 100 cited articles on rheumatoid arthritis: A bibliometric analysis. Medicine.

[21] Abhishek A., Doherty M., Kuo C.F., Mallen C.D., Zhang W., Grainge M.J. (2017). Rheumatoid arthritis is getting less frequent-results of a nationwide population-based cohort study. Rheumatology.

[22] Myasoedova E., Davis J.M., Achenbach S.J., Matteson E.L., Crowson C.S. (2019). Trends in Prevalence of Functional Disability in Rheumatoid Arthritis Compared with the General Population. Mayo Clin. Proc..

[23] Scott I.C., Mount J., Barry J., Kirkham B. (2020). Factors associated with disability in patients with rheumatoid arthritis with persistent moderate disease activity: A retrospective cohort study. BMC Rheumatol..

[24] Hsieh P., Wu O., Geue C., McIntosh E., McInnes I.B., Siebert S. (2020). Economic burden of rheumatoid arthritis: A systematic review of literature in biologic era. Ann. Rheum. Dis..

[25] Gaitonde P., Shaya F.T. (2016). Economic and Productivity Consequences Associated with Rheumatoid Arthritis Among Non-Institutionalized Individuals in The United States. Res. Methods–Cost Methods.

[26] Kwon H., Kim Y.L., Lee S.M. (2015). Relation between functional ability and health-related quality of life of children with juvenile rheumatoid arthritis. J. Phys. Ther. Sci..

[27] Hefti F. (2015). Juvenile rheumatoid arthritis. Pediatric Orthopedics in Practice.

[28] https://www.rheumresearch.org/.

[29] Gaujoux-Viala C., Gossec L., Cantagrel A., Dougados M., Fautrel B., Mariette X., Nataf H., Saraux A., Trope S., Combe B. (2014). Recommendations of the French Society for Rheumatology for managing rheumatoid arthritis. Jt. Bone Spine Rev. Rhum..

[30] Ly J., Gow P., Dalbeth N. (2007). Colchicine prescribing and safety monitoring in patients with gout. N. Z. Med. J..

[31] Firestein G.S. (2003). Evolving concepts of rheumatoid arthritis. Nature.

[32] Rudan I., Sidhu S., Papana A., Meng S.J., Xin-Wei Y., Wang W., Campbell-Page R.M., Demaio A.R., Nair H., Sridhar D. (2015). Global Health Epidemiology Reference Group (GHERG). Prevalence of rheumatoid arthritis in low- and middle-income countries: A systematic review and analysis. J. Glob. Health.

[33] Van Vollenhoven R.F. (2009). Sex differences in rheumatoid arthritis: More than meets the eye. BMC Med..

[34] Gerosa M., de Angelis V., Riboldi P., Meroni P. (2008). Rheumatoid Arthritis: A Female Challenge. Women’s Health.

[35] Kanaan S.B., Onat O.E., Balandraud N., Azzouz D.F., Roudier J., Ozcelik T., Lambert N.C. (2013). Does telomere shortening in women with rheumatoid arthritis predict x chromosome inactivation bias?. Ann. Rheum. Dis..

[36] Charukevič G., Miltinienė D., Dadonienė J. (2021). Mortality in Patients with Rheumatoid Arthritis: A Retrospective Cohort Study and Systematic Review. Med. Sci. Forum.

[37] Lee Y., Ahn G.Y., Lee J., Shin J., Lee T., Park D.J., Song Y.J., Kim M.K., Bae S. (2021). Excess mortality persists in patients with rheumatoid arthritis. Int. J. Rheum. Dis..

[38] Widdifield J., Paterson J.M., Huang A., Bernatsky S. (2018). Causes of Death in Rheumatoid Arthritis: How Do They Compare to the General Population?. Arthritis Care Res..

[39] Almutairi K.B., Inderjeeth C.A., Preen D.B., Keen H.I., Nossent J.C. (2023). Mortality Trends Among Patients with Rheumatoid Arthritis in Western Australia. Rheumatol. Ther..

[40] Arend W.P., Dayer J.M. (1995). Inhibition of the production and effects of interleukin-1 and tumor necrosis factor α in rheumatoid arthritis. Arthritis Rheum..

[41] Furst D.E., Breedveld F.C., Kalden J.R., Smolen J.S., Burmester G.R., Bijlsma J.W., Dougados M., Emery P., Keystone E.C., Klareskog L. (2004). Updated consensus statement on biological agents, specifically tumour necrosis factor α (TNFα) blocking agents and interleukin-1 receptor antagonist (IL-1ra), for the treatment of rheumatic diseases, 2004. Ann. Rheum. Dis..

[42] Holdsworth E.A., Donaghy B., Fox K.M., Desai P., Collier D.H., Furst D.E. (2021). Biologic and Targeted Synthetic DMARD Utilization in the United States: Adelphi Real World Disease Specific Programme for Rheumatoid Arthritis. Rheumatol. Ther..

[43] Harrington R., Al Nokhatha S.A., Conway R. (2020). JAK Inhibitors in Rheumatoid Arthritis: An Evidence-Based Review on the Emerging Clinical Data. J. Inflamm. Res..

[44] Yip R.M.L., Yim C.W. (2021). Role of Interleukin 6 Inhibitors in the Management of Rheumatoid Arthritis. J. Clin. Rheumatol..

[45] Du F.H., Mills E.A., Mao-Draayer Y. (2017). Next-generation anti-CD20 monoclonal antibodies in autoimmune disease treatment. Auto Immun. Highlights.

[46] Massalska M., Maslinski W., Ciechomska M. (2020). Small Molecule Inhibitors in the Treatment of Rheumatoid Arthritis and Beyond: Latest Updates and Potential Strategy for Fighting COVID-19. Cells.

[47] Radu A.F., Bungau S.G. (2021). Management of Rheumatoid Arthritis: An Overview. Cells.

[48] Singh J.A., Saag K.G., Bridges S.L., Akl E.A., Bannuru R.R., Sullivan M.C., Vaysbrot E., McNaughton C., Osani M., Shmerling R.H. (2016). 2015 American college of rheumatology guideline for the treatment of rheumatoid arthritis. Arthritis Rheumatol..

[49] Moura M.D.G., Lopes L.C., Silva M.T., Barberato-Filho S., Motta R.H.L., Bergamaschi C.C. (2018). Use of steroid and nonsteroidal anti-inflammatories in the treatment of rheumatoid arthritis: Systematic review protocol. Medicine.

[50] Kvien T.K., Balsa A., Betteridge N., Buch M.H., Durez P., Favalli E.G., Favier G., Gabay C., Geenen R., Gouni-Berthold I. (2020). Considerations for improving quality of care of patients with rheumatoid arthritis and associated comorbidities. RMD Open.

[51] Fassbender H.G. (2008). The clinical presentation of rheumatoid arthritis: The results from three separate pathogenetic mechanisms in adults and children. Acta Clin. Croat..

[52] Ibrahim F., Ma M., Scott D.L., Scott I.C. (2022). Defining the relationship between pain intensity and disease activity in patients with rheumatoid arthritis: A secondary analysis of six studies. Arthritis Res. Ther..

[53] Kronzer V.L., Davis J.M. (2021). Etiologies of Rheumatoid Arthritis: Update on Mucosal, Genetic, and Cellular Pathogenesis. Curr. Rheumatol. Rep..

[54] Klockars M., Koskela R.S., Järvinen E., Kolari P.J., Rossi A. (1987). Silica exposure and rheumatoid arthritis: A follow up study of granite workers 1940–81. Br. Med. J. (Clin. Res. Ed.).

[55] Khanna S., Jaiswal K.S., Gupta B. (2017). Managing Rheumatoid Arthritis with Dietary Interventions. Front. Nutr..

[56] Tiwari V., Jandu J.S., Bergman M.J. (2022). Rheumatoid Factor.

[57] Holm B.E., Sandhu N., Tronstrom J., Lydolph M., Trier N.H., Houen G. (2015). Species cross-reactivity of rheumatoid factors and implications for immunoassays. Scand. J. Clin. Lab. Investig..

[58] Gehin J.E., Klaasen R.A., Norli E.S., Warren D.J., Syversen S.W., Goll G.L., Bjøro T., Kvien T.K., Mjaavatten M.D., Bolstad N. (2021). Rheumatoid factor and falsely elevated results in commercial immunoassays: Data from an early arthritis cohort. Rheumatol. Int..

[59] Romão V.C., Fonseca J.E. (2021). Etiology and Risk Factors for Rheumatoid Arthritis: A State-of-the-Art Review. Front. Med..

[60] Van Boxel J.A., Paget S.A. (1975). Predominantly T-cell infiltrate in rheumatoid synovial membranes. N. Engl. J. Med..

[61] Morimoto C., Romain P.L., Fox D.A., Anderson P., DiMaggio M., Levine H., Schlossman S.F. (1988). Abnormalities in CD4+ T-lymphocyte subsets in inflammatory rheumatic diseases. Am. J. Med..

[62] Simon A.K., Seipelt E., Sieper J. (1994). Divergent T-cell cytokine patterns in inflammatory arthritis. Proc. Natl. Acad. Sci. USA.

[63] Ziolkowska M., Koc A., Luszczykiewicz G., Ksiezopolska-Pietrzak K., Klimczak E., Chwalinska-Sadowska H., Maslinski W. (2000). High levels of IL-17 in rheumatoid arthritis patients: IL-15 triggers in vitro IL-17 production via cyclosporin A-sensitive mechanism. J. Immunol..

[64] Philippou E., Nikiphorou E. (2018). Are we really what we eat? Nutrition and its role in the onset of rheumatoid arthritis. Autoimmun. Rev..

[65] Demoruelle M.K., Deane K.D., Holers V.M. (2014). When and where does inflammation begin in rheumatoid arthritis?. Curr. Opin. Rheumatol..

[66] López-Mejías R., Carmona F.D., Genre F., Remuzgo-Martínez S., González-Juanatey C., Corrales A., Vicente E.F., Pulito-Cueto E.V., Miranda-Filloy J.A., Ramírez Huaranga M.A. (2019). Identification of a 3′-Untranslated Genetic Variant of RARB Associated with Carotid Intima-Media Thickness in Rheumatoid Arthritis: A Genome-Wide Association Study. Arthritis Rheumatol..

[67] Scherer H.U., Häupl T., Burmester G.R. (2020). The etiology of rheumatoid arthritis. J. Autoimmun..

[68] Nielen M.M.J., van Schaardenburg D., Reesink H.W., van de Stadt R.J., van der Horst-Bruinsma I.E., de Koning M.H.M.T., Habibuw M.R., Vandenbroucke J.P., Dijkmans B.A.C. (2004). Specific autoantibodies precede the symptoms of rheumatoid arthritis: A study of serial measurements in blood donors. Arthritis Rheum..

[69] Ao Y., Wang Z., Hu J., Yao M., Zhang W. (2023). Identification of essential genes and immune cell infiltration in rheumatoid arthritis by bioinformatics analysis. Sci. Rep..

[70] Ota M., Nagafuchi Y., Hatano H., Ishigaki K., Terao C., Takeshima Y., Yanaoka H., Kobayashi S., Okubo M., Shirai H. (2021). Dynamic landscape of immune cell-specific gene regulation in immune-mediated diseases. Cell.

[71] Yamada S., Nagafuchi Y., Wang M., Ota M., Hatano H., Takeshima Y., Okubo M., Kobayashi S., Sugimori Y., Masahiro N. (2023). Immunomics analysis of rheumatoid arthritis identified precursor dendritic cells as a key cell subset of treatment resistance. Rheum. Dis..

[72] Smolen J.S., Aletaha D., Barton A., Burmester G.R., Emery P., Firestein G.S., Arthur Kavanaugh A., Iain B. (2018). McInnes, I.B.; Solomon, D.H.; et al. Rheumatoid arthritis. Nat. Rev. Dis. Primers.

[73] Mucke J., Krusche M., Burmester G.R. (2022). A broad look into the future of rheumatoid arthritis. Ther. Adv. Musculoskelet. Dis..

[74] Edrees A.F., Misra S.N., Abdou N.I. (2005). Anti-tumor necrosis factor (TNF) therapy in rheumatoid arthritis: Correlation of TNF-alpha serum level with clinical response and benefit from changing dose or frequency of infliximab infusions. Clin. Exp. Rheumatol..

[75] Duong S.Q., Crowson C.S., Athreya A., Atkinson E.J., Davis J.M., Warrington K.J., Matteson E.L., Weinshilboum R., Wang L., Myasoedova E. (2022). Clinical predictors of response to methotrexate in patients with rheumatoid arthritis: A machine learning approach using clinical trial data. Arthritis Res. Ther..

[76] Haraoui B., Bykerk V. (2007). Etanercept in the treatment of rheumatoid arthritis. Ther. Clin. Risk Manag..

[77] Feist E., Baraliakos X., Behrens F., Thaçi D., Klopsch T., Plenske A., Blindzellner L.K., Klaus P., Meng T., Löschmann P.A. (2022). Effectiveness of Etanercept in Rheumatoid Arthritis: Real-World Data from the German Non-interventional Study ADEQUATE with Focus on Treat-to-Target and Patient-Reported Outcomes. Rheumatol. Ther..

[78] Stacy J.M., Greenmyer J.R., Beal J.R., Sahmoun A.E., Diri E. (2021). The efficacy of low dose short-term prednisone therapy for remission induction in newly diagnosed rheumatoid arthritis patients. Adv. Rheumatol..

[79] Guadagnin D.A., Mazzali L.V., Skare T.L., Kahlow B.S. (2021). Treating rheumatoid arthritis with leflunomide monotherapy versus combination therapy with methotrexate. Eur. J. Rheumatol..

[80] Smolen J.S., Landewé R.B.M., Bergstra S.A., Kerschbaumer A., Sepriano A., Aletaha D., Caporali R., Edwards C.J., Hyrich K.L., Pope J.E. (2023). EULAR recommendations for the management of rheumatoid arthritis with synthetic and biological disease-modifying antirheumatic drugs: 2022 update. Ann. Rheum. Dis..

[81] Tanaka Y. (2022). Subcutaneous injection of methotrexate: Advantages in the treatment of rheumatoid arthritis. Mod. Rheumatol..

[82] Brown P.M., Pratt A.G., Isaacs J.D. (2016). Mechanism of action of methotrexate in rheumatoid arthritis, and the search for biomarkers. Nat. Rev. Rheumatol..

[83] Conway R., Carey J.J. (2017). Risk of liver disease in methotrexate treated patients. World J. Hepatol..

[84] Bedoui Y., Guillot X., Sélambarom J., Guiraud P., Giry C., Jaffar-Bandjee M.C., Ralandison S., Gasque P. (2019). Methotrexate an Old Drug with New Tricks. Int. J. Mol. Sci..

[85] Downey C. (2016). Serious infection during etanercept, infliximab and adalimumab therapy for rheumatoid arthritis: A literature review. Int. J. Rheum. Dis..

[86] Singh J.A., Cameron C., Noorbaloochi S., Cullis T., Tucker M., Christensen R., Ghogomu E.T., Coyle D., Clifford T., Tugwell P. (2015). Risk of serious infection in biological treatment of patients with rheumatoid arthritis: A systematic review and meta-analysis. Lancet.

[87] Dixon W.G. (2015). Rheumatoid arthritis: Biological drugs and risk of infection. Lancet.

[88] Scheinfeld N. (2005). Adalimumab: A review of side effects. Expert Opin. Drug Saf..

[89] Kingsbury D.J., Bader-Meunier B., Patel G., Arora V., Kalabic J., Kupper H. (2014). Safety, effectiveness, and pharmacokinetics of adalimumab in children with polyarticular juvenile idiopathic arthritis aged 2 to 4 years. Clin. Rheumatol..

[90] Hanoodi M., Mittal M. (2023). Methotrexate.

[91] Bashar T., Apu M.N.H., Mostaid M.S., Islam M.S., Hasnat A. (2018). Pharmacokinetics and bioavailability study of a prednisolone tablet as a single oral dose in Bangladeshi healthy volunteers. Dose Response.

[92] Yasir M., Goyal A., Sonthalia S. (2022). Corticosteroid Adverse Effects.

[93] Breedveld F.C., Dayer J.M. (2000). Leflunomide: Mode of action in the treatment of rheumatoid arthritis. Ann. Rheum. Dis..

[94] Strand V., Cohen S., SchiV M., Weaver A., Fleischmann R., Cannon G., Fox R., Moreland L., Olsen N., Furst D. (1999). Treatment of active rheumatoid arthritis with leflunomide compared to placebo and methotrexate. Arch. Intern Med..

[95] Smolen J.S., Kalden J.R., Scott D.L., Rozman B., Kvien T.K., Loew-Friedrich I., Oed C., Rosenburg R. (1999). Efficacy and safety of leflunomide compared with placebo and sulphasalazine in active rheumatoid arthritis: A double-blind, randomised, multicentre trial. Lancet.

[96] Chong A.S., Huang W., Liu W., Luo J., Shen J., Xu W., Ma L., Blinder L., Xiao F., Xu X. (1999). In vivo activity of leflunomide: Pharmacokinetic analyses and mechanism of immunosuppression. Transplantation.

[97] Cain D., Cidlowski J. (2017). Immune regulation by glucocorticoids. Nat. Rev. Immunol..

[98] Furman B.L. (2019). Prednisolone. Ref. Modul. Biomed. Sci..

[99] Huscher D., Thiele K., Gromnica-Ihle E., Hein G., Demary W., Dreher R., Zink A., Buttgereit F. (2009). Dose-related patterns of glucocorticoid-induced side effects. Ann. Rheum. Dis..

[100] Scott D.L., Strand V., Strand V., Scott D.L., Simon L.S. (1997). Leflunomide: A new immunosuppressive drug. Novel Therapeutic Agents for the Treatment of Autoimmune Diseases.

[101] Hambleton P., McMahon S. (1990). Drug actions on delayed-type hypersensitivity in rats with developing and established adjuvant arthritis. Agents Actions.

[102] Bartlett R.R. (1986). Immunopharmacological profile of HWA 486, a novel isoxazol derivative—II. In vivo immunomodulating eVectsdiVer from those of cyclophosphamide, prednisolone, or cyclosporin A. Int. J. Immunopharmacol..

[103] Kuo E.A., Hambleton P.T., Kay D.P., Evans P.L., Matharu S.S., Little E., McDowall N., Jones C.B., Hedgecock C.J., Yea C.M. (1996). Synthesis, structure—Activity relationships, and pharmacokinetic properties of dihydroorotate dehydrogenase inhibitors: 2-cyano-3-cyclopropyl-3- hydroxy-N-[3′-methyl-4′-(trifluoromethyl)phenyl]propenamide and related compounds. J. Med. Chem..

[104] Xu X., Blinder L., Shen J., Gong H., Finnegan A., Williams J.W., Chong A.S. (1997). In vivo mechanism by which leflunomide controls lymphoproliferative and autoimmune disease in MRL/MpJ-Ipr/Ipr mice. J. Immunol..

[105] Elder R.T., Xu X., Williams J.W., Gong H., Finnegan A., Chong A.S.F. (1997). The immunosuppressive metabolite of leflunomide, A77 1726, a Vects murine T cells through two biochemical mechanisms. J. Immunol..

[106] Watanabe R., Okano T., Gon T., Yoshida N., Fukumoto K., Yamada S., Hashimoto M. (2022). Difficult-to-treat rheumatoid arthritis: Current concept and unsolved problems. Front. Med..

[107] Nagy G., Roodenrijs N.M.T., Welsing P.M., Kedves M., Hamar A., van der Goes M.C., Kent A., Bakkers M., Blaas E., Senolt L. (2021). EULAR definition of difficult-to-treat rheumatoid arthritis. Ann. Rheum. Dis..

[108] Lindler B.N., Long K.E., Taylor N.A., Lei W. (2020). Use of Herbal Medications for Treatment of Osteoarthritis and Rheumatoid Arthritis. Medicines.

[109] Siemoneit U., Koeberle A., Rossi A., Dehm F., Verhoff M., Reckel S., Maier T.J., Jauch J., Northoff H., Bernhard F. (2010). Inhibition of microsomal prostaglandin E2 synthase-1 as a molecular basis for the anti-inflammatory actions of boswellic acids from frankincense. Br. J. Pharm..

[110] Safayhi H., Mack T., Sabieraj J., Anazodo M.I., Subramanian L.R., Ammon H.P. (1992). Boswellic acids: Novel, specific, nonredox inhibitors of 5-lipoxygenase. J. Pharm. Exp. Ther..

[111] Majeed M., Majeed S., Narayanan N.K., Nagabhushanam K. (2019). A pilot, randomized, double-blind, placebo-controlled trial to assess the safety and efficacy of a novel Boswellia serrata extract in the management of osteoarthritis of the knee. Phytother. Res..

[112] Majeed M., Vaidyanathan P., Natarajan S., Majeed S., Vuppala K.K. (2016). Effect of Boswellin^®^ Super on knee pain in Japanese adults: A randomized, double-blind, placebo-controlled trial. Eur. J. Biomed..

[113] Chopra A., Lavin P., Patwardhan B., Chitre D. (2000). Randomized double blind trial of an ayurvedic plant derived formulation for treatment rheumatoid arthritis. J. Rheumatol..

[114] Razavi S.Z.E., Karimi M., Kamalinejad M. (2019). The efficacy of topical oliban oil (*Boswellia carterii*, B.) in relieving the symptoms of knee osteoarthritis. Phys. Med. Rehabil. Electrodiagn..

[115] Goel A., Kunnumakkara A.B., Aggarwal B.B. (2008). Curcumin as “Curecumin”: From kitchen to clinic. Biochem. Pharmacol..

[116] Chin K.-Y. (2016). The spice for joint inflammation: Anti-inflammatory role of curcumin in treating osteoarthritis. Drug Des. Dev. Ther..

[117] Prasad S., Gupta S.C., Tyagi A.K., Aggarwal B.B. (2014). Curcumin, a component of golden spice: From bedside to bench and back. Biotechnol. Adv..

[118] Aggarwal B.B., Surh Y.-J., Shishodia S. (2007). The Molecular Targets and Therapeutic Uses of Curcumin in Health and Disease.

[119] Shep D., Khanwelkar C., Gade P., Karad S. (2020). Efficacy and safety of combination of curcuminoid complex and diclofenac versus diclofenac in knee osteoarthritis. Medicine.

[120] Kuptniratsaikul V., Dajpratham P., Taechaarpornkul W., Buntragulpoontawee M., Lukkanapichonchut P., Chootip C., Saengsuwan J., Tantayakom K., Laongpech S. (2014). Efficacy and safety of Curcuma domestica extracts compared with ibuprofen in patients with knee osteoarthritis: A multicenter study. Clin. Interv. Aging.

[121] Shep D., Khanwelkar C., Gade P., Karad S. (2019). Safety and efficacy of curcumin versus diclofenac in knee osteoarthritis: A randomized open-label parallel-arm study. Trials.

[122] Shoara R., Hashempur M.H., Ashraf A., Salehi A., Dehshahri S., Habibagahi Z. (2015). Efficacy and safety of topical *Matricaria chamomilla* L. (chamomile) oil for knee osteoarthritis: A randomized controlled clinical trial. Complement. Ther. Clin. Pract..

[123] El Mihyaoui A., Esteves da Silva J.C.G., Charfi S., Candela Castillo M.E., Lamarti A., Arnao M.B. (2022). Chamomile (*Matricaria chamomilla* L.): A Review of Ethnomedicinal Use, Phytochemistry and Pharmacological Uses. Life.

[124] Pirouzpanah S., Mahboob S., Sanayei M., Hajaliloo M., Safaeiyan A. (2017). The effect of chamomile tea consumption on inflammation among rheumatoid arthritis patients: Randomized clinical trial. Prog. Nutr..

[125] Yamagata K. (2020). Protective effect of epigallocatechin gallate on endothelial disorders in atherosclerosis. J. Cardiovasc. Pharmacol..

[126] Karatas A., Dagli A.F., Orhan C., Gencoglu H., Ozgen M., Sahin N., Sahin K., Koca S.S. (2020). Epigallocatechin 3-gallate attenuates arthritis by regulating Nrf2, HO-1, and cytokine levels in an experimental arthritis model. Biotechnol. Appl. Biochem..

[127] Srirangan S., Choy E.H. (2010). The role of interleukin 6 in the pathophysiology of rheumatoid arthritis. Ther. Adv. Musculoskelet. Dis..

[128] Bouhlali E.d.T., Hmidani A., Bourkhis B., Khouya T., Ramchoun M., Filali-Zegzouti Y., Alem C. (2020). Phenolic profile and anti-inflammatory activity of four Moroccan date (*Phoenix dactylifera* L.) seed varieties. Heliyon.

[129] Zahin M., Ahmad I., Gupta R.C., Aqil F. (2014). Punicalagin and Ellagic Acid Demonstrate Antimutagenic Activity and Inhibition of Benzo[a]pyrene Induced DNA Adducts. Biomed Res. Int..

[130] Lee C.J., Chen L.G., Liang W.L., Wang C.C. (2010). Anti-inflammatory effects of *Punica granatum* Linne in vitro and in vivo. Food Chem..

[131] Ghavipour M., Sotoudeh G., Tavakoli E., Mowla K., Hasanzadeh J., Mazloom Z. (2017). Pomegranate extract alleviates disease activity and some blood biomarkers of inflammation and oxidative stress in Rheumatoid Arthritis patients. Eur. J. Clin. Nutr..

[132] Zhang W., Dai S.-M. (2012). Mechanisms involved in the therapeutic effects of *Paeonia lactiflora* Pallas in rheumatoid arthritis. Int. Immunopharmacol..

[133] Zhang L., Wei W. (2020). Anti-inflammatory and immunoregulatory effects of paeoniflorin and total glucosides of paeony. Pharmacol. Ther..

[134] Delazar A., Sarker S.D., Nahar L., Jalali S.B., Modarresi M., Hamedeyazdan S., Babaei H., Javadzadeh Y., Asnaashari S., Moghadam S.B. (2013). Rhizomes of *Eremostachys laciniata*: Isolation and Structure Elucidation of Chemical Constituents and a Clinical Trial on Inflammatory Diseases. Adv. Pharm. Bull..

[135] Erdemoglu N., Turan N.N., Cakõcõ I., Şener B., Aydõn A. (2006). Antioxidant activities of some Lamiaceae plant extracts. Phytother. Res..

[136] Tan S., Xu J., Lai A., Cui R., Bai R., Li S., Liang W., Zhang G., Jiang S., Liu S. (2019). Curculigoside exerts significant anti–arthritic effects in vivo and in vitro via regulation of the JAK/STAT/NF–κB signaling pathway. Mol. Med. Rep..

[137] Han J., Wan M., Ma Z., Hu C., Yi H. (2020). Prediction of targets of curculigoside a in osteoporosis and rheumatoid arthritis using network pharmacology and experimental verification. Drug Des. Dev. Ther..

[138] Pyatt D.W., Yang Y., Mehos B., Le A., Stillman W., Irons R.D. (2000). Hematotoxicity of the Chinese Herbal Medicine *Tripterygium wilfordii* Hook f in CD34-Positive Human Bone Marrow Cells. Mol. Pharm..

[139] Tang W., Zuo J.-P. (2012). Immunosuppressant discovery from *Tripterygium wilfordii* Hook f: The novel triptolide analog (5R)-5-hydroxytriptolide (LLDT-8). Acta Pharm. Sin..

[140] Cibere J., Deng Z., Lin Y., Ou R., He Y., Wang Z., Thorne A., Lehman A.J., Tsang I.K., Esdaile J.M. (2003). A randomized double blind, placebo controlled trial of topical *Tripterygium wilfordii* in rheumatoid arthritis: Reanalysis using logistic regression analysis. J. Rheumatol..

[141] Yu D.Y. (1983). Clinical observation of 144 cases of rheumatoid arthritis treated with glycoside of Radix *Tripterygium wilfordii*. J. Tradit. Chin. Med..

[142] Guo J.L., Yuan S.X., Wang X.C., Xu S.X., Li D.D. (1981). *Tripterygium wilfordii* Hook f in rheumatoid arthritis and ankylosing spondylitis. Preliminary report. Chin. Med. J..

[143] Tao X.L., Sun Y., Dong Y., Xiao Y.L., Hu D.W., Shi Y.P., Zhu Q.L., Dai H., Zhang N.Z. (1989). A prospective, controlled, double-blind, cross-over study of *Tripterygium wilfodii* hook F in treatment of rheumatoid arthritis. Chin. Med. J..

[144] Liu X., Wang Z., Qian H., Tao W., Zhang Y., Hu C., Mao W., Guo Q. (2022). Natural medicines of targeted rheumatoid arthritis and its action mechanism. Front. Immunol..

[145] Liu W., Qian X., Ji W., Lu Y., Wei G., Wang Y. (2016). Effects and safety of sinomenine in treatment of rheumatoid arthritis contrast to methotrexate: A systematic review and meta-analysis. J. Tradit. Chin. Med..

[146] Zeng M.Y., Tong Q.Y. (2020). Anti-inflammation effects of sinomenine on macrophages through suppressing activated TLR4/NF-κB signaling pathway. Curr. Med. Sci..

[147] Tong B., Yu J., Wang T., Dou Y., Wu X., Kong L., Dai Y., Xia Y. (2015). Sinomenine suppresses collagen-induced arthritis by reciprocal modulation of regulatory T cells and Th17 cells in gut-associated lymphoid tissues. Mol. Immunol..

[148] Feng Z.T., Yang T., Hou X.Q., Wu H.Y., Feng J.T., Ou B.J., Cai S.-J., Li J., Mei Z.-G. (2019). Sinomenine mitigates collagen-induced arthritis mice by inhibiting angiogenesis. BioMed. Pharmacother..

[149] Zhao X., Kim Y.-R., Min Y., Zhao Y., Do K., Son Y.-O. (2021). Natural Plant Extracts and Compounds for Rheumatoid Arthritis Therapy. Medicina.

[150] Sun H., Wang M., Zhang A., Ni B., Dong H., Wang X. (2013). UPLC-Q-TOF-HDMS analysis of constituents in the root of two kinds of Aconitum using a metabolomics approach. Phytochem. Anal..

[151] Luo Y., Liu M., Xia Y., Dai Y., Chou G., Wang Z. (2010). Therapeutic effect of norisoboldine, an alkaloid isolated from radix linderae, on collagen-induced arthritis in mice. Phytomedicine.

[152] Wei Z.F., Tong B., Xia Y.F., Lu Q., Chou G.X., Wang Z.T., Dai Y. (2013). Norisoboldine suppresses osteoclast differentiation through preventing the accumulation of TRAF6-TAK1 complexes and activation of MAPKs/NF-kappaB/c-Fos/NFATc1 pathways. PLoS ONE.

[153] Wei Z.F., Jiao X.L., Wang T., Lu Q., Xia Y.F., Wang Z.T., Guo Q.-L., Chou G.-X., Dai Y. (2013). Norisoboldine alleviates joint destruction in rats with adjuvant-induced arthritis by reducing RANKL, IL-6, PGE(2), and MMP-13 expression. Acta Pharmacol. Sin..

[154] Hilvo M., Baranauskiene L., Salzano A.M., Scaloni A., Matulis D., Innocenti A., Scozzafava A., Monti S.M., Di Fiore A., De Simone G. (2008). Biochemical characterization of CA IX, one of the most active carbonic anhydrase isozymes. J. Biol. Chem..

[155] Tian Y., Maosheng Q., Wei X. (2010). Experimental Study of GuizhiShaoyaoZhimu Decoction on Gene Regulation of Synovial Cell Apoptosis in Rheumatoid Arthritis. Contemp. Med..

[156] Guo Q., Mao X., Zhang Y., Meng S., Xi Y., Ding Y., Zhang X., Dai Y., Liu X., Wang C. (2016). Guizhi-Shaoyao-Zhimu decoction attenuates rheumatoid arthritis partially by reversing inflammation-immune system imbalance. J. Transl. Med..

[157] Feng C., Chen R., Wang K., Wen C., Xu Z. (2021). Chinese traditional medicine (GuiZhi-ShaoYao-ZhiMu decoction) as an add-on medication to methotrexate for rheumatoid arthritis: A meta-analysis of randomized clinical trials. Ther. Adv. Chronic Dis..

[158] Mbizo J., Okafor A., Sutton M.A., Burkhart E.N., Stone L.M. (2016). Complementary and Alternative Medicine Use by Normal Weight, Overweight, and Obese Patients with Arthritis or Other Musculoskeletal Diseases. J. Altern. Complement. Med..

[159] Van de Laar M. (2012). Pain Treatment in Arthritis-Related Pain: Beyond NSAIDs. Open Rheumatol. J..

[160] Marrelli M., Amodeo V., Perri M.R., Conforti F., Statti G. (2020). Essential Oils and Bioactive Components against Arthritis: A Novel Perspective on Their Therapeutic Potential. Plants.

[161] Choudhary M., Kumar V., Malhotra H., Singh S. (2015). Medicinal plants with potential anti-arthritic activity. J. Intercult. Etnopharmacol..

[162] Soeken K.L., Miller S.A., Ernst E. (2003). Herbal medicines for the treatment of rheumatoid arthritis: A systematic review. Rheumatology.

[163] Ahmed S., Anuntiyo J., Malemud C.J., Haqqi T.M. (2005). Biological basis for the use of botanicals in osteoarthritis and rheumatoid arthritis: A review. Evid. Based Complement. Altern. Med..

[164] Boneva B., Marchev A., Amirova K., Ganova P., Georgiev M., Tchorbanov A., Mihaylova N. (2023). Crocus sativus Extract as a Biological Agent for Disease-Modifying Therapy of Collagenase-Induced Mouse Model of Osteoarthritis. Life.

[165] Wang Y., Chen S., Du K., Liang C., Wang S., Owusu Boadi E., Li J., Pang X., He J., Chang Y. (2021). Traditional herbal medicine: Therapeutic potential in rheumatoid arthritis. J. Ethnopharmacol..

[166] Wang D.A., Williams C.G., Yang F., Elisseeff J.H. (2004). Enhancing the tissue-biomaterial interface: Tissue-initiated integration of biomaterials. Adv. Funct. Mater..

[167] Federici T., Boulis N. (2007). Gene therapy for peripheral nervous system diseases. Curr. Gene Ther..

[168] Sazani P., Vacek M.M., Kole R. (2002). Short-term and long-term modulation of gene expression by antisense therapeutics. Curr. Opin. Biotechnol..

[169] Gopi C., Dhanaraju M.D., Dhanaraju K. (2022). Antisense oligonucleotides: Recent progress in the treatment of various diseases. Beni-Suef Univ. J. Basic Appl. Sci..

[170] Makalish T.P., Golovkin I.O., Oberemok V.V., Laikova K.V., Temirova Z.Z., Serdyukova O.A., Novikov I.A., Rosovskyi R.A., Gordienko A.I., Zyablitskaya E.Y. (2021). Anti-Rheumatic Effect of Antisense Oligonucleotide Cytos-11 Targeting TNF-α Expression. Int. J. Mol. Sci..

[171] Wijesinghe S.N., Lindsay M.A., Jones S.W. (2021). Oligonucleotide Therapies in the Treatment of Arthritis: A Narrative Review. Biomedicines.

[172] Nakazawa M., Ishii H., Aono H., Takai M., Honda T., Aratani S., Fukamizu A., Nakamura H., Yoshino S., Kobata T. (2001). Role of Notch-1 intracellular domain in activation of rheumatoid synoviocytes. Arthritis Rheum..

[173] Stanford S.M., Maestre M.F., Campbell A.M., Bartok B., Kiosses W.B., Boyle D.L., Arnett H.A., Mustelin T., Firestein G.S., Bottini N. (2013). Protein tyrosine phosphatase expression profile of rheumatoid arthritis fibroblast-like synoviocytes: A novel role of SH2 domain-containing phosphatase 2 as a modulator of invasion and survival. Arthritis Rheum..

[174] Pearson M.J., Jones S.W. (2016). Review: Long Noncoding RNAs in the Regulation of Inflammatory Pathways in Rheumatoid Arthritis and Osteoarthritis. Arthritis Rheumatol..

[175] Lian W.S., Ko J.Y., Wu R.W., Sun Y.C., Chen Y.S., Wu S.L., Weng L.H., Jahr H., Wang F.S. (2018). MicroRNA-128a represses chondrocyte autophagy and exacerbates knee osteoarthritis by disrupting Atg12. Cell Death Dis..

[176] Borgonetti V., Galeotti N. (2021). Intranasal delivery of an antisense oligonucleotide to the RNA-binding protein HuR relieves nerve injury-induced neuropathic pain. Pain.

[177] Mohan A., Fitzsimmons B., Zhao H.T., Jiang Y., Mazur C., Swayze E.E., Kordasiewicz H.B. (2018). Antisense oligonucleotides selectively suppress target RNA in nociceptive neurons of the pain system and can ameliorate mechanical pain. Pain.

[178] Luo X., Fitzsimmons B., Mohan A., Zhang L., Terrando N., Kordasiewicz H., Ji R.R. (2018). Intrathecal administration of antisense oligonucleotide against p38O± but not p38OI MAP kinase isoform reduces neuropathic and postoperative pain and TLR4-induced pain in male mice. Brain Behav. Immun..

[179] Schett G., Tanaka Y., Isaacs J.D. (2021). Why remission is not enough: Underlying disease mechanisms in RA that prevent cure. Nat. Rev. Rheumatol..

[180] Alexander T., Thiel A., Rosen O., Massenkeil G., Sattler A., Kohler S., Mei H., Radtke H., Gromnica-Ihle E.G., Arnold R. (2009). Depletion of autoreactive immunologic memory followed by autologous hematopoietic stem cell transplantation in patients with refractory SLE induces long-term remission through de novo generation of a juvenile and tolerant immune system. Blood.

[181] Hsieh M.C., Lee J.J. (2018). Preliminary study of VR and AR applications in medical and healthcare education. J. Nurs. Health Stud..

[182] Guo Q., Wang Y., Xu D., Nossent J., Pavlos N.J., Xu J. (2018). Rheumatoid arthritis: Pathological mechanisms and modern pharmacologic therapies. Bone Res..

[183] Kim J., Chun K., McGowan J., Zhang Y., Czernik P.J., Mell B., Joe B., Chattopadhyay S., Holoshitz J., Ritu Chakravarti R. (2021). 14-3-3ζ: A suppressor of inflammatory arthritis. Proc. Natl. Acad. Sci. USA.

[184] Zimmerman D.H., Mikecz K., Markovics A., Carambula R.E., Ciemielewski J.C., Toth D.M., Glant T.T., Rosenthal K.S. (2021). Vaccination by two DerG LEAPS conjugates incorporating distinct proteoglycan (PG, aggrecan) epitopes provides therapy by different immune mechanisms in a mouse model of rheumatoid arthritis. Vaccines.

[185] Mun S., Lee J., Park M., Shin J., Lim M.K., Kang H.G. (2021). Serum biomarker panel for the diagnosis of rheumatoid arthritis. Arthritis Res. Ther..

[186] Tao W., Concepcion A.N., Vianen M., Marijnissen A.C.A., Lafeber F.P.G.J., Radstake T.R.D.J., Pandit A. (2021). Multiomics and machine learning accurately predict clinical response to adalimumab and etanercept therapy in patients with rheumatoid arthritis. Arthritis Rheumatol..

[187] Bressem K.K., Vahldiek J.L., Adams L., Niehues S.M., Haibel H., Rodriguez V.R., Torgutalp M., Protopopov M., Proft F., Rademacher J. (2021). Deep learning for detection of radiographic sacroiliitis: Achieving expert-level performance. Arthritis Res. Ther..

[188] Vodencarevic A., Tascilar K., Hartmann F., Reiser M., Hueber A.J., Haschka J., Bayat S., Meinderink T., Knitza J., Mendez L. (2021). Advanced machine learning for predicting individual risk of flares in rheumatoid arthritis patients tapering biologic drugs. Arthritis Res. Ther..

[189] Sagner M., McNeil A., Puska P., Auffray C., Price N.D., Hood L., Lavie C.J., Han Z.-G., Chen Z., Brahmachari S.K. (2017). The P4 health spectrum—A predictive, preventive, personalized and participatory continuum for promoting healthspan. Prog. Cardiovasc. Dis..

[190] Zhao J., Guo S., Schrodi S.J., He D. (2022). Cuproptosis and cuproptosis–related genes in rheumatoid arthritis: Implication, prospects, and perspectives. Front. Immunol. Sec. Autoimmune Autoinflammatory Disord..

[191] Wu W., Qin M., Jia W., Huang Z., Li Z., Yang D., Huang M., Xiao C., Long F., Mao J. (2019). Cystathionine-γ-lyase ameliorates the histone demethylase JMJD3-mediated autoimmune response in rheumatoid arthritis. Cell. Mol. Immunol..

[192] Zhu M., Ding Q., Lin Z., Fu R., Zhang F., Li Z., Zhang M., Zhu Y. (2023). New Targets and Strategies for Rheumatoid Arthritis: From Signal Transduction to Epigenetic Aspect. Biomolecules.

[193] Ben Mrid R., Bouchmaa N., Ainani H., El Fatimy R., Malka G., Mazini L. (2022). Anti-rheumatoid drugs advancements: New insights into the molecular treatment of rheumatoid arthritis. Biomed. Pharmacother..

[194] Nicholson T.A., Sagmeister M., Wijesinghe S.N., Farah H., Hardy R.S., Jones S.W. (2023). Oligonucleotide Therapeutics for Age-Related Musculoskeletal Disorders: Successes and Challenges. Pharmaceutics.

